# Genetic interactions: the missing links for a better understanding of cancer susceptibility, progression and treatment

**DOI:** 10.1186/1476-4598-7-4

**Published:** 2008-01-10

**Authors:** Christopher A Maxwell, Víctor Moreno, Xavier Solé, Laia Gómez, Pilar Hernández, Ander Urruticoechea, Miguel Angel Pujana

**Affiliations:** 1 Bioinformatics and Biostatistics Unit, and Translational Research Laboratory, Catalan Institute of Oncology, IDIBELL, Gran Vía km 2.7, L'Hospitalet 08907, Barcelona, Spain

## Abstract

It is increasingly clear that complex networks of relationships between genes and/or proteins govern neoplastic processes. Our understanding of these networks is expanded by the use of functional genomic and proteomic approaches in addition to computational modeling. Concurrently, whole-genome association scans and mutational screens of cancer genomes identify novel cancer genes. Together, these analyses have vastly increased our knowledge of cancer, in terms of both "part lists" and their functional associations. However, genetic interactions have hitherto only been studied in depth in model organisms and remain largely unknown for human systems. Here, we discuss the importance and potential benefits of identifying genetic interactions at the human genome level for creating a better understanding of cancer susceptibility and progression and developing novel effective anticancer therapies. We examine gene expression profiles in the presence and absence of co-amplification of the 8q24 and 20q13 chromosomal regions in breast tumors to illustrate the molecular consequences and complexity of genetic interactions and their role in tumorigenesis. Finally, we highlight current strategies for targeting tumor dependencies and outline potential matrix screening designs for uncovering molecular vulnerabilities in cancer cells.

## Background

Most of the current knowledge of cancer susceptibility, progression and treatment has been generated by traditional approaches, in which small numbers of genes or proteins are characterized in depth to study the molecular mechanisms of neoplastic processes. With the advent of large-scale functional genomic and proteomic ("omic") methodologies, additional mechanistic insights into neoplasia have been uncovered. Whole-genome association studies for cancer risk variants and somatic mutation screening projects have completed their initial phases and will provide the "part lists" of cancer genes, both at the germline [1] and the somatic levels [2]. Transcript analyses have identified expression profiles that provide accurate prognoses for cancer patients [3]. Systematic mapping of protein-protein interactions is currently being carried out in what are referred to as 'interactome' mapping projects. This research will elucidate the wiring diagram of protein associations in cells [4,5]. These types of genes and/or protein (gene/protein) functional relationships can be modeled together to provide better understanding and predict molecular mechanisms of neoplasia [6-10].

Genetic interactions are identified when the action of one gene measured through a molecular, cellular or organism phenotype is modified by one or more other genes. They provide insight into biological processes that are complementary but which frequently do not overlap with other types of gene/protein associations [11]. To date, genetic interactions remain largely unknown on a large scale in human systems. Previous reviews and assays gave excellent descriptions of the role of genetic interactions in understanding phenotypic variability [12-14]. Here, we focus our discussion on the potential of studying human genetic interactions as a means of not only better understanding cancer susceptibility and progression but also, and most importantly, developing novel anticancer treatments.

## Discussion

### A step forward in cancer research

The use of 'model organisms' (i.e. species that meet the criteria of being representative of particular organisms or cellular behaviour) has played a prominent role in all aspects of biology. Since the first description of the complete sequence of an eukaryotic genome, the yeast *Saccharomyces cerevisiae *[15], the use of model organisms has grown with the development of high-throughput screenings for large-scale genomic and proteomic investigations [16]. Using these technologies and models, researchers have been able to acquire a greater understanding of how most, if not all, genes/proteins act coordinately to determine the properties of an organelle, cell or organism [8]. This "systems-level" research is also being used to decipher complex networks of molecular relationships in human systems, in healthy or normal conditions and their perturbation in disease or unconventional molecularly – or environmentally-modified conditions.

Of the possible gene-to-gene relationships in complex networks, genetic interactions were first mapped on a large scale in yeast [17]. These studies identified "synthetic lethal" interactions that occur when the combination of two gene deletions causes cell death whereas neither deletion is lethal by itself (i.e. non-essential genes). In addition to "synthesis", interactions can also be revealed by "epistasis", which is commonly used to define "genetic interactions" in statistical terms and describes the deviation from additivity for a quantitative phenotype by the effect of genetic variants or mutations at different loci [18-20]. Many models of interactions between different loci have been identified, including the combination of "aggravating" or agonistic and "alleviating" or buffering relationships [21,22].

Given the complexity of genetic interaction relationships, understanding the topology of genetic interaction networks is crucial for establishing genotype-phenotype correlations. This knowledge has clear implications in the study of certain aspects of common, "complex" or non-Mendelian diseases, such as the incidence of cancer in the general population, where the sum of minor gene effects contributes to the risk [23]. In addition to germline cancer risk, the mapping of genetic interactions in humans may also provide information on cancer progression and treatment, due to the large number of genes involved and the complexity of pathological phenotypes. Lessons learned from the study of genetic interactions in model organisms could be applied in defining hitherto unimaginable approaches for studying complex biological aspects of neoplastic processes.

### Lessons from model organisms

The study of genetic interactions has been typically used in the annotation of signaling or metabolic pathways and protein complexes as a means of inferring the logical order of their components and examining pathway cross-talk [24]. A remarkable finding made in early systematic deletion analysis studies of yeast indicated that most eukaryotic genes are dispensable for viability. Only ~20% of *Saccharomyces cerevisiae *genes are essential for haploid cells grown in standard laboratory conditions [25,26].

Topological analysis of the yeast genetic interaction network has revealed the importance of gene interactions in phenotype modelling. More than 30 interactions on average were identified for non-essential genes and five-fold more for essential genes [17,27]. Accordingly, a recent estimate predicts ~200,000 synthetic lethal interactions in the global yeast genetic network [12]. When extrapolated to humans, these figures result in a vast number of genetic interactions with a specific phenotype potentially influenced by hundreds of different gene combinations. This estimate does not include combinations of more than two genes, which remain difficult to calculate at this stage. These predictions for phenotype determinants in humans clearly reveal the need for large-scale genetic interaction projects to provide more in-depth knowledge of complex diseases. Beyond the examination of single or small numbers of genes, systems-level analyses of model organisms have also uncovered the complex structure of genetic interaction networks. Epistasis extends beyond individual genes to clusters or modules of functionally related genes [28]. At this systems-level, perturbing one functional module can have aggravating or alleviating consequences on another.

Further advances could potentially be made through computational prediction of human genetic interactions using different types of experimentally generated molecular interactions. However, the modest overlap observed between experimentally identified genetic interactions and protein-protein interactions in yeast suggests that any predictions will render largely incomplete data [11,29-31]. Furthermore, given a pair of genes and their corresponding gene products, calculations in yeast give approximately four times more genetic interactions than protein-protein interactions. Finally, using orthologs to transfer genetic interactions across species may show even lower confidence than for protein-protein interologs [32], due to the observed complexity of the relationships between apparently molecularly unrelated signaling or metabolic pathways [28,33]. Taken together, computational predictions of human genetic interactions may provide lists of candidate gene pairs but, until further advances are made, they will require extensive experimental validation.

Systematic identification of genetic interactions in a multicellular organism has been described in a recent study by Lehner et al. [34]. The authors identified genetic interactions in *Caenorhabditis elegans *using the RNA interference (RNAi) method, which entails analyzing hypomorphic alleles rather than complete transcript depletion, which is the preferred methodology in the majority of yeast studies. Lehner et al. [34] highlighted the global relevance to phenotype modeling of genetic interactions between components of the same pathway, rather than interactions between components of different pathways. This study also anticipated the methodological problems and interpretational caveats that may appear when performing large-scale screening of genetic interactions in humans. Human studies require vast amounts of effort, the prevention of false positives in large-scale RNAi screens [35], and the incorporation of multiple designs to account for different cellular models and conditions [14]. Nevertheless, we believe that they can have an enormous impact on our knowledge of cancer.

### Genetic interactions and cancer susceptibility

Projects such as the Cancer Genetic Markers of Susceptibility (CGEMS) initiative and the work carried out by Cancer Research UK within an international consortium are currently providing the preliminary partial lists of low-penetrance genetic variants for risk of different cancer types in the general population [36,37]. Initial analyses of this data have used a whole-genome approach to identify the main effects of individual variants. However, earlier studies based on candidate-gene approaches had already highlighted the existence of interactions between variants in different genes influencing cancer susceptibility [38]. It is now thought that the analysis of large series of individuals, together with the development of novel statistical approaches, will facilitate the assessment of millions of variant combinations which will, in turn, enable us to elucidate the impact of genetic interactions in cancer susceptibility [39]. Promising analyses based on simulations and empirical data have demonstrated that the likelihood of detecting significant associations increases when epistasis is taken into account [39-41].

The drawback of analyses taking into account epistasis is the trade-off between statistical power and false discovery rate. Current whole genome association studies consider over 500,000 single nucleotide polymorphisms, and *P *values need to be under 10^-7 ^to account for multiple comparisons. Since low-penetrance alleles provide modest increases in risk, in the range of 20–30%, the sample sizes needed to detect main effects are in the range of 2,000 to 5,000 cases and a similar number of controls. When the aim is to detect gene interactions, the required number of tests increases to 2.5 × 10^11 ^and the *P *value should be corrected to 2 × 10^-13^. The sample sizes needed for these significance levels are larger, but currently feasible – in the range of 6,000 to 15,000 cases for variants with high frequencies (20–40%) [41,42]. Novel statistical approaches have been proposed which reduce the dimensionality of the problem of searching for relevant gene interactions. The multifactor dimensionality reduction (MDR) method has been successfully applied to limited-scale scenarios [38,43,44]. Other promising proposals include the restricted partition method (RPM) [45] and combinatorial searching methods (CSM) [46]. Nevertheless, given the large number of expected false positive genetic interactions that will be proposed following analysis of whole-genome association studies, it will be necessary to carry out extensive validation in multiple populations and biological models based on the experimental identification of genetic interactions [47].

### Genetic interactions and cancer progression

Cancer arises from the consecutive acquisition of genetic alterations that, in general, produce the loss of function or transcriptional down-regulation of tumor suppressor genes and the activation or transcriptional up-regulation of oncogenes [48]. Downstream of the genetic alterations in tumorigenesis we find expression changes in many genes, which lead to abnormal regulation of biological processes such as the cell cycle and apoptosis [9,49]. Consequently, it is thought that genetic and molecular alterations promote tumorigenesis in the context of highly connected and regulated gene/protein networks [7,8,50]. Thus, the progression from normal epithelium to benign dysplasia to metastases is relatively well characterized for some cancer types and highlights a specific molecular program [51]. The importance of studying genetic interactions between tumor suppressor genes, such as the retinoblastoma *RB1 *gene, and oncogenes, such as the *RAS *family, was established in earlier works that provided fundamental insights into the mechanisms of tumorigenesis and metastasis [52-54].

**Figure 1:**
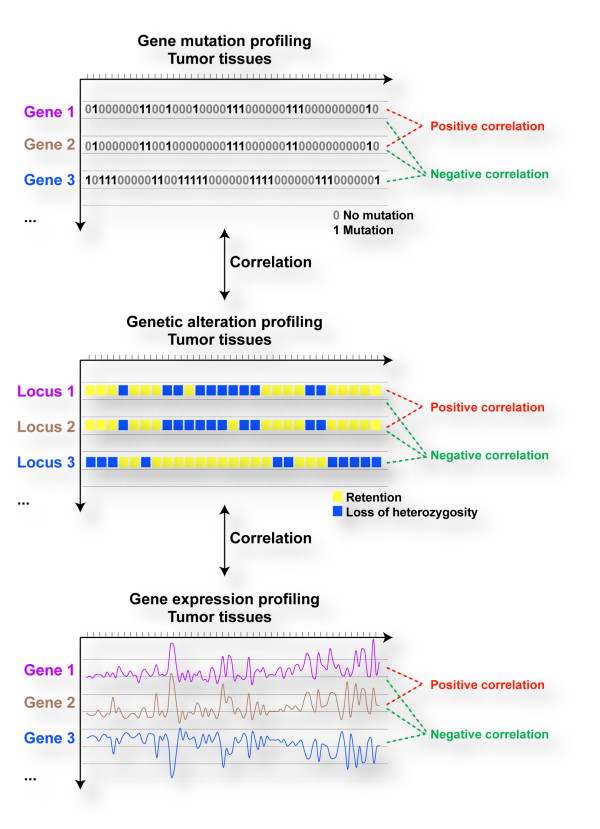
Correlation of genetic and genomic alterations and transcript profiles across hundreds of tumors may reveal gene/protein functional dependencies that are useful for predicting genetic interactions of interest for cancer progression and anticancer therapies.

Knowledge of the sequence of genetic alterations that contribute to the neoplastic process could be used to target cancer cells according to the concept of synthetic lethality. Thus, it may be possible to reveal cancer genetic alterations useful for identifying genetic interactions by examining the profiles of somatic mutations, genomic alterations or gene expression changes in large series of tumors (Fig. 1). Under this hypothesis, the examination of highly correlated profiles may help to detect strong gene/protein dependencies in cancer cells. A greater understanding of these dependencies may illuminate mechanisms of carcinogenesis with insights into prognosis. Differences in biological meanings could also be extrapolated from positive versus negative correlations, implying molecular dependencies in different directions. Research into this hypothesis will be aided by the characterization at different genomic levels of large series of tumors. The Cancer Genome Project at the Sanger Institute has sequenced hundreds of human genes in tumors, tissues and cell lines [55,56]. Recent results obtained by other groups have demonstrated that oncogene activation in tumors is usually mutually exclusive. However, it has also been shown that some co-occurring mutations may reflect dependencies that are critical for neoplasia [57]. The identification of these types of neoplastic-specific functional dependencies could improve prognosis prediction and tumor classification, while targeting these dependencies may enhance therapeutic efficacy.

### Empirical analysis of genetic interactions acting on tumor phenotypes

The integrative analysis of cancer molecular data sheds light on the mechanisms of neoplasia [6,10,58-60]. As discussed above, as large series of tumors and cell lines are characterized we will gradually be able to detect molecular dependencies and the ways in which they influence cancer progression. Co-amplification of specific genomic regions in breast tumors has been suggested and may represent cancer gene interactions between oncogenes that promote neoplasia. Specifically, co-amplification of the 8q24 and 20q13 regions may target the *c-MYC *and *ZNF217 *or *BCAS1 *proto-oncogenes, respectively [60].

**Figure 2:**
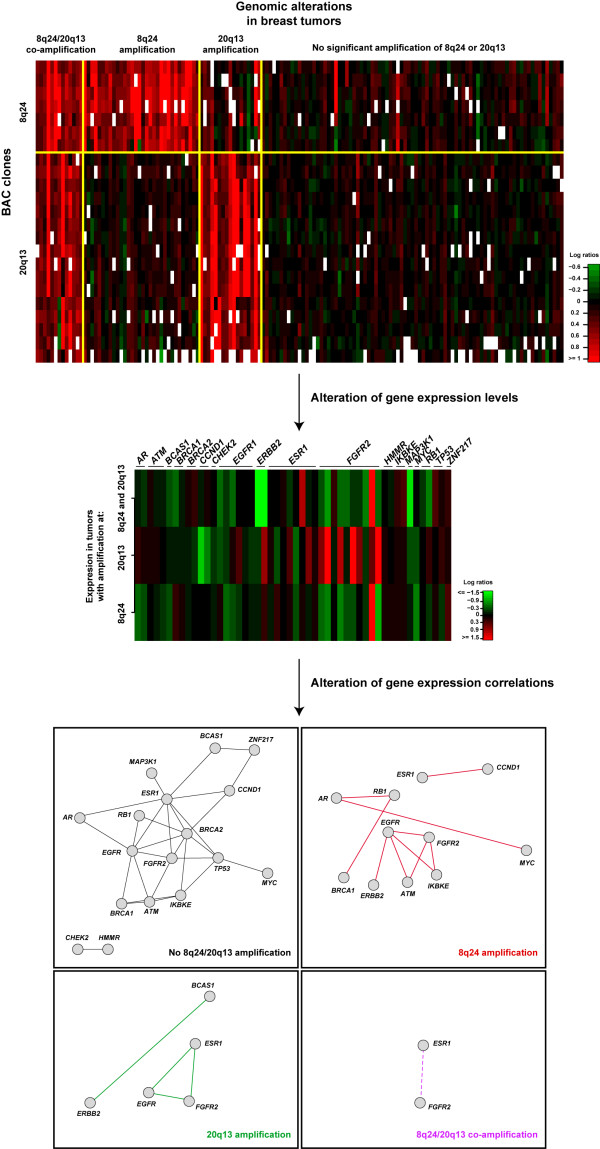
Empirical analysis of genetic interactions acting on tumorigenesis. Pre-processed and normalized data were taken from Chin et al. [60]. White squares represent missing data. Log expression ratios are relative to tumors that do not show amplification at 8q24 or 20q13. Average expression values are shown for each gene probe (not detailed) in each tumor set (8q24/20q13, 8q24 or 20q13 amplification). Transcript correlations were calculated using the Pearson correlation coefficient and adjusted *P *values with a false discovery rate of 5%; only significant correlations with *Q *values < 0.05 are shown, except for the 8q24/20q13 co-amplification set (dashed line; Q ~0.07).

To assess the 8q24/20q13 interaction, we evaluated molecular phenotypes such as transcript levels and co-expression between known breast cancer tumor suppressors and oncogenes (Fig. 2). In the absence of emergent genetic interactions, one may expect co-amplification of 8q24/20q13 to produce no relevant differences in gene expression between breast tumors. However, differences were observed beyond the simple additivity of expression levels for many genes when comparing tumors showing co-amplification with tumors showing single region amplification or no amplification; for example, *ERBB2 *showed marked down-regulation in tumors with co-amplification while the other tumors showed higher expression levels (Fig. 2, middle panel). Molecular phenotypic alterations also produced increases or decreases in correlation values of gene expression profiles, which are comparable to the concepts of alleviating or aggravating interactions; for example, a cluster of significant correlations between *ATM*,*EGFR*, *ERBB2*, *FGFR2 *and *IKBKE *appeared to be stronger in tumors with only 8q24 amplifications (Fig. 2, bottom panel). Portraits of expression correlations were noticeably different between tumors with different genomic alterations, to the extent that in co-amplification there were no significant correlations. These observations suggest differences in the molecular mechanisms that promote cancer progression determined by genetic interactions between cancer genes. This analysis is limited by the sample size series and could be extended by using multivariate analyses. Nevertheless, it illustrates the potential usefulness of genetic interactions in understanding tumor phenotypes.

### Genetic interactions and cancer treatment

Robustness, which describes the ability of cells or organisms to maintain viability and functionality despite (multiple) molecular perturbations, is a fundamental principle in many biological systems [50,61-63]. Tumors are robust systems *par excellence *and it is thought that robustness is sustained by functional redundancy maintained by cell heterogeneity and feedback transcriptional control mechanisms [50]. However, cancer robustness might be offset by the extreme fragility of molecular networks. This idea is the basis for the hypothesis that uncommon perturbations of regular neoplastic processes would have dramatic effects on cancer cells [64].

Information on the acquisition of germline and somatic genetic alterations that contribute to neoplasia could then be systematically analyzed to find the Achilles' heel of cancer cells. The authors of a landmark study that paved the way for this type of strategy discovered that cells deficient in the breast cancer tumor suppressors BRCA1 and BRCA2 are completely dependent on the normal activity of the poly (ADP-ribose) polymerase family member 1 (PARP1) [65,66]. Inherited germline mutations in *BRCA1 *or *BRCA2 *genes confer high risk of breast and ovarian cancer, always accompanied by the somatic inactivation of the remaining wild-type allele [67-69]. Consequently, BRCA1/BRCA2-deficient tumor cells show genetic and genomic instability due in part to impaired DNA damage repair [70]. Against this background, depletion of PARP1 causes synthetic lethality due to severe defective homologous recombination necessary for regular DNA repair activity. Clinical trials using PARP1 inhibitors for breast cancer, and other malignancies characterized by DNA repair defects, now explore the potential of these interventions.

The *BRCA/PARP1 *paradigm could be applied in different terms to the observed tumor resistance to anticancer therapies. An example of this would be the use of trastuzumab for treating breast cancer with *ERBB2 *amplifications, which represent ~25% of all cases [71]. Although these tumors show great dependence on the HER-2 receptor for growth and, as a result, frequently respond to trastuzumab treatment, they almost always develop resistance. Genetic interaction screens performed in the presence of trastuzumab, or blocking HER-2, may reveal those genes whose inactivation leads to synthetic lethality or sickness pattern in breast cancer cells. Similar observations could be made using imatinib and *KIT *gene mutations in gastrointestinal stromal tumors [72] or the Philadelphia chromosome in chronic myeloid leukaemia [73]. A landmark study has recently demonstrated the usefulness of the concept of synthetic lethality in identifying genes that, when depleted, increase the sensitivity of cancer cells to paclitaxel [74]. Another recent study has revealed the co-activation of receptor tyrosine kinases as a mechanism that allows cancer cells to survive single compound-based treatments [75]. These observations further support the use of combined target therapies based on the depletion of selected genes to achieve superior therapeutic efficacy.

### Genetic interactions screen designs

**Figure 3:**
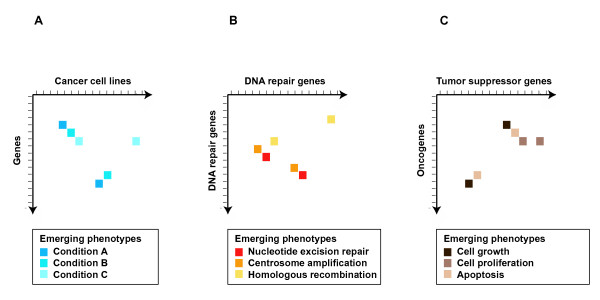
Examples of matrix screens of genetic interactions of interest in studying cancer susceptibility, progression and treatment: (A) Interactions are identified in standard cancer cell lines by depleting one gene at a time and through quantitation of emerging phenotypes; (B) Interactions are identified between genes involved in the DNA repair processes, with quantitation of emerging phenotypes highlighting repair subcategories; and (C) Interactions are revealed between known tumor suppressor genes and oncogenes, with quantitation of emerging phenotypes highlighting different properties of the neoplastic process.

The fundamental aim of whole-genome systematic screens of human genetic interactions is to reveal multiple new anticancer therapy alternatives. Among various possible designs, these screens could be performed with a matrix format and standard cancer cell lines and by depleting genes using siRNAs or viral-packed shRNA constructs [76,77] (Fig. 3A). This approach would uncover a cancer genetic interaction network without *a priori *hypotheses concerning gene function, although current knowledge of genetic alterations in cancer cell lines should help to identify promising targets. This design could be extended following the suggestion made by Whitehurst et al. [74], in which sublethal concentrations of anticancer agents are used.

Different interpretations of genetic interactions and their applications will depend on whether the studied cell lines or models represent dominant or recessive inherited cancer. Genetic interactions in dominant inherited cancer syndromes should specifically target tumor cells that, for instance, lack both autosomal copies of a tumor suppressor gene. When targeting these tumors with synthetic lethal interventions, normal tissue and cells should remain unaffected unless dose effects appear due to the inactivation of a single allele in all cells. In recessive inherited cancer syndromes such as Fanconi anemia, in which all cells are inactivated for a specific gene, therapeutic targeting with synthetic lethal disruption could potentially act systemically, depending on cell-type specificities.

Designs for matrix screens of genetic interactions can also be based on the current knowledge of common molecular alterations in neoplasia (Fig. 3B). Many human genes encoding for proteins involved in DNA damage repair processes are mutated or epigenetically altered in cancer cells [78]. Currently, there are ~150 human genes annotated with DNA repair-related Gene Ontology terms [79]. Mapping interactions between these genes, including somatic or germline mutations such as the mismatch defects observed in colorectal tumors [80], is therefore expected to be beneficial in different cancer types. Again, the suggestion of Whitehurst et al. [74] could be applied to the identification of interactions between DNA repair genes by administering DNA-damaging agents at sublethal concentrations. In addition, many of these genes have orthologs in model organisms that could be used to study the conservation of synthetic lethality relationships and their role in altering the cellular response to DNA damage.

Another possible matrix strategy that could be used to identify new treatments consists of mapping interactions between known tumor suppressor genes and oncogenes (Fig. 3C). Unlike "passenger alterations", genetic alterations that contribute to neoplasia occur in a specific combination and order between tumor suppressor genes and oncogenes [48,49]. This observation, otherwise fragility of cancer genetic networks, could be useful in the identification of therapeutic targets. This matrix design combines the over-expression of tumor suppressors and the down-regulation or depletion of oncogenes. The experimental designs presented here are intended to stimulate interest in the utility and importance of this type of gene relationship in different areas of cancer research. We are confident that future research can and will lead to more sophisticated experimental designs for mapping genetic interactions in human models, which will greatly enhance our understanding of the molecular mechanisms of cancer.

## Conclusion

Interactions between human genes are largely unknown. From studies in model organisms, it is clear that genetic interactions at the human genome-scale need to be identified in order to better understand common human diseases, with cancer being the paradigm. The detection of these interactions will be invaluable to our understanding of cancer risk, by suggesting hypotheses concerning the molecular mechanisms of susceptibility; of cancer progression, by revealing gene/protein functional dependencies; and of cancer treatment, by providing the knowledge required to develop new, efficient anticancer strategies.

## Authors' contributions

XS, VM and MAP designed and performed the empirical data analysis. VM performed the simulation of association data. CAM and MAP drafted the manuscript. VM, LG, PH, AU helped to draft the manuscript. MAP conceived the article. All authors read and approved the final manuscript.
